# Mitochondrial Fusion Proteins and Human Diseases

**DOI:** 10.1155/2013/293893

**Published:** 2013-05-27

**Authors:** Michela Ranieri, Simona Brajkovic, Giulietta Riboldi, Dario Ronchi, Federica Rizzo, Nereo Bresolin, Stefania Corti, Giacomo P. Comi

**Affiliations:** ^1^Dino Ferrari Centre, Neuroscience Section, Department of Pathophysiology and Transplantation (DEPT), University of Milan, Neurology Unit, IRCCS Foundation Ca' Granda Ospedale Maggiore Policlinico, Via F. Sforza 35, 20122 Milan, Italy; ^2^IRCCS Eugenio Medea, Bosisio Parini, 23842 Lecco, Italy

## Abstract

Mitochondria are highly dynamic, complex organelles that continuously alter their shape, ranging between two opposite processes, fission and fusion, in response to several stimuli and the metabolic demands of the cell. Alterations in mitochondrial dynamics due to mutations in proteins involved in the fusion-fission machinery represent an important pathogenic mechanism of human diseases. The most relevant proteins involved in the mitochondrial fusion process are three GTPase dynamin-like proteins: mitofusin 1 (MFN1) and 2 (MFN2), located in the outer mitochondrial membrane, and optic atrophy protein 1 (OPA1), in the inner membrane. An expanding number of degenerative disorders are associated with mutations in the genes encoding MFN2 and OPA1, including Charcot-Marie-Tooth disease type 2A and autosomal dominant optic atrophy. While these disorders can still be considered rare, defective mitochondrial dynamics seem to play a significant role in the molecular and cellular pathogenesis of more common neurodegenerative diseases, for example, Alzheimer's and Parkinson's diseases. This review provides an overview of the basic molecular mechanisms involved in mitochondrial fusion and focuses on the alteration in mitochondrial DNA amount resulting from impairment of mitochondrial dynamics. We also review the literature describing the main disorders associated with the disruption of mitochondrial fusion.

## 1. Introduction

Mitochondrial fusion and fission are fundamental processes underlying cellular dynamics [1]. They are closely related, and therefore any alterations in their equilibrium may lead to disease. How the impairment of these pathways leads to neurological dysfunction and neurodegeneration is still largely debated. Fusion allows the exchange of contents, DNA, and metabolites between neighboring mitochondria, including damaged or senescent mitochondria, promoting their survival [2, 3]. Fission is necessary for proper mitochondrial transport, which depends on the specific energy demands of subcellular regions. Fission also regulates apoptosis through segregation of the most critically injured mitochondria [1, 4]. Dynamin-related protein 1 (DRP1), a cytosolic dynamin-related GTPase, plays a central role in fission by promoting mitochondrial division through its oligomerization into multimeric spiral structures [5]. To trigger mitochondrial fission, DRP1 must be recruited to the mitochondrial outer membrane, where several molecules of unknown functions colocalize; among them, mitochondrial fission 1 and mitochondrial fission factor have been proposed to be involved in DRP1 recruitment, although recent *in vitro* studies seem to not support this hypothesis [6, 7]. 

Fusion of the outer mitochondrial membrane depends on two GTPase family members: mitofusin 1 (MFN1) and mitofusin 2 (MFN2). MFN2 is also present in the endoplasmic reticulum, controlling its morphology and facilitating mitochondrial calcium influx from endoplasmic reticulum stores [8]. Optic atrophy protein 1 (OPA1) mediates fusion of the inner mitochondrial membrane and is involved in maintaining mitochondrial inner-membrane potential and controlling apoptosis; its downregulation leads to aberrant cristae remodeling and to the release of cytochrome *c* [9–13].

These proteins take part in the fusion pathway in two consecutive steps. Initially, the dimerization of mitofusins results in the tethering of the outer membranes of adjoining mitochondria. Before fusion, curving of the outer membranes is promoted by the phospholipase D-dependent hydrolysis of cardiolipin. In the second step, fusion of the inner membranes requires a motor-like process driven by OPA1 and coordinated by various other proteins, including the prohibitins. This basic two-step process has been confirmed in most mammalian cells, although its regulation and the repertoire of specific accessory proteins are likely to be highly context dependent. A detailed description of this mechanism can be found in [14] and is reviewed in [15].

**Figure 1:**
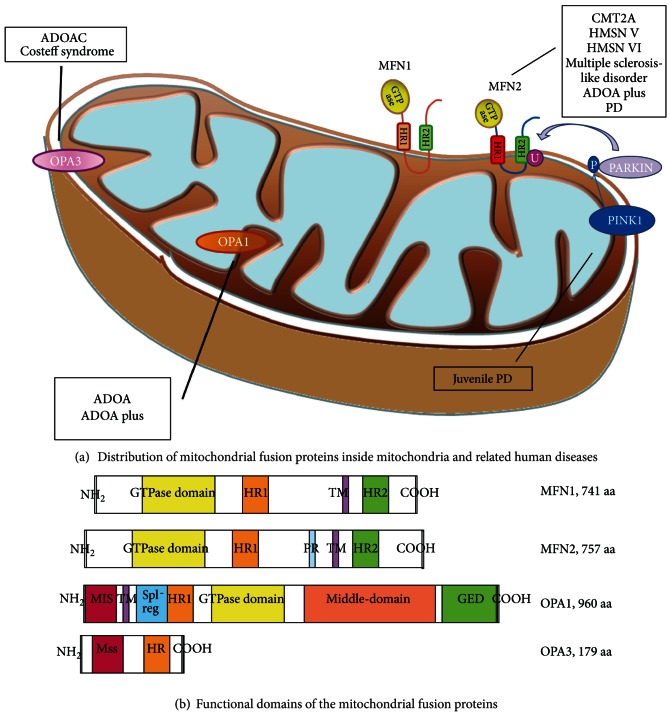
Distribution of mitochondrial fusion proteins inside mitochondria and related human diseases resulting from defects in gene expression or protein function (a). Mitofusin 2 (MFN2) and optic atrophy protein 3 (OPA3) are present in the outer mitochondrial membrane; dynamin-related protein optic atrophy 1 (OPA1) is localized in the intermembrane space and is tethered to the inner mitochondrial membrane. The top right of the figure illustrates the molecular pathway responsible for mitophagy: PTEN-induced putative kinase protein 1 (PINK1) phosphorylates Parkin, an ubiquitin E3 ligase that targets several outer membrane proteins including mitofusin. Ubiquitination of MFN2 in damaged mitochondria starts the mitophagic process. ADOA: autosomal dominant optic atrophy; ADOAC: autosomal dominant optic atrophy and cataract; HR: heptad repeat; PD: Parkinson's disease. Below (b), schematics of functional domains of the mitochondrial fusion proteins (HR: heptad repeat domain; TM: transmembrane domain; PR: proline-rich domain; MIS: mitochondrial import sequence; GED: GTPase effector domain; Mss: mitochondrial signal sequence).

Recently, mutations in *OPA1* and *MFN2* have been described in patients with autosomal dominant optic atrophy (ADOA) and multisystem clinical involvement (including progressive external ophthalmoplegia (PEO)) who harbored mitochondrial DNA (mtDNA) deletions in skeletal muscle [16–18] (Table 1, Figure 1(a)). Furthermore, heterozygous *MFN2* mutations have been associated with mtDNA depletion [19], suggesting that mitochondrial fusion defects not only impair mtDNA maintenance, but may also affect mtDNA replication. Indeed, as recently described in yeast mitochondria by Hori et al., the fusion event facilitates recombination-mediated mtDNA replication and prevents the generation of cells with reduced mtDNA copy number [20].

Downregulation of *MFN2* expression has also been observed in nonneurological disorders such as metabolic diseases (obesity and diabetes), vascular proliferative disorders [21–23], and cardiomyopathy. Although further studies are required, *in vivo* studies seem to indicate various degrees of impairment in cardiac function, ranging from moderate hypertrophy reported in *MFN2* knockout mice to severe heart failure in the absence of both mitofusins [24, 25]. OPA1 aberrant expression has also been associated with late-onset cardiomyopathy in mouse models carrying a heterozygous null mutation in *OPA1* [26].

Another protein with predominantly mitochondrial localization, OPA3, is involved in two allelic disorders: a dominantly inherited optic atrophy associated with early-onset cataracts and an autosomal recessive progressive neurodegenerative disease with 3-methylglutaconic aciduria, named Costeff syndrome [27, 28]. OPA3 produces two distinct RNA transcripts, OPA3A and OPA3B. OPA3A is highly expressed, conserved among different species, and its loss plays a role in disease etiology, while OPA3B is expressed at lower levels and its mutation has not been associated with human disease [29]. Despite the similarity of clinical phenotypes associated with alterations in OPA3A and OPA1, the underlying pathogenic mechanisms still require elucidation.

## 2. Mitochondrial Fusion Genes and Related Proteins

The first description of the process of mitochondrial fusion was given in yeast by the end of 1960 [30], but mitochondrial dynamics were later analyzed in mammalian cellular models as well [31–33]. The discovery of the first gene encoding a protein mediator of mitochondrial fusion was made in *Drosophila melanogaster* [34]; this protein, fuzzy onions, is a homolog of the human mitofusins. Other molecules involved in the fusion machinery have recently been described, including Mgm1 in *Saccharomyces cerevisiae*, which was identified as a homolog of human OPA1 [35]. Similarly, DLP1/DRP1 and other proteins required for mitochondrial fission were first studied in yeast (Dnm1p) [36] and in *Caenorhabditis elegans* (DRP-1) [37], with description of the mammalian homologues following soon after [38, 39].

These *in vivo* studies suggested that the core requirements for mitochondrial fusion are similar in yeast and mammalian cells. This early evidence has recently been confirmed by *in vitro* fusion assays employing isolated yeast and mammalian mitochondria in which mitochondrial proteins are fluorescently tagged or revealed by chemiluminescent reactions, allowing investigators to trace the fates of mixed mitochondria. Appropriate centrifugation steps and temperature gradients are commonly used to promote initial tethering of mitochondrial membranes and their fusion [40, 41]. These approaches do not always permit the discrimination of fusion events occurring at the outer and inner membranes, which involve specific and distinct sets of proteins.

In humans, MFN1 (741 residues) and MFN2 (757 residues) are two large nuclear-encoded dynamin-like GTPases that have both their N-terminus and C-terminus exposed to the cytosol. Mitofusins harbor a GTPase domain close to the N-terminus that is involved in the GTP hydrolysis required for the oligomerization of the mitofusins (homo- and heterotypic oligomers) and promotes fusion of the outer membranes of adjacent mitochondria [23, 49]. MFN2 also includes a proline-rich domain involved in protein-protein interactions.

Ubiquitination and subsequent degradation of the mitofusins were demonstrated to be critical in yeast, probably because they allow steric hindrance to be overcome [50, 51]. The Mdm30-dependent ubiquitination of mitofusins in yeasts and flies is a driving force that promotes the late stages of fusion, after which the GTP hydrolysis indispensable for this posttranslational modification can occur. Mdm30 also mediates mitofusin turnover. In vertebrates, ubiquitination of the mitofusins leads to their extraction from the membrane by p97 and their degradation by proteasomes. This process, recently described as the PTEN-induced kinase 1 (PINK1)/Parkin pathway, occurs in damaged or senescent mitochondria, preventing their ability to rejoin the mitochondrial network before final clearance via autophagy [52].

The GTPase domain usually consists of five motifs (G1–G5), although this occurrence has not been completely tested in the mitofusins. Motif G1 binds the phosphate of the GTP molecule, motif G3 coordinates the Mg^2+^ needed for hydrolysis, motifs G1–G3 form the catalytic center, and motifs G4 and G5 provide the specific conformation for GTP binding (not ATP) [23]. Two hydrophobic heptad repeat domains (HR) are localized in the middle (HR1) and C-terminal (HR2) regions and provide the basis for most coiled-coil interactions (Figure 1(b)). The HR2 domain was shown to be of great importance for mitochondrial fusion activity; formation of HR2 dimers promotes the generation of a mitochondrial tether before mitochondrial fusion [49, 53]. Mutations in the GTPase domain and mutations in the HR2 region affect the fusion activity of the mitofusins. Further, as reported by Huang and colleagues [54], DRP1 seems to be involved in facilitating MFN2-mediated fusion by interfering with interactions between the two HR domains, but the exact role of DRP1 in mitochondrial fusion remains elusive.

OPA1  consists of 30 coding exons spread over 100 kb of genomic DNA. The encoded product contains an N-terminal mitochondrial localization sequence that is responsible for importing the protein into the mitochondrial inner membrane. This region also has three putative cleavage sites for the mitochondrial processing peptidase, which removes the mitochondrial localization sequence upon import into mitochondria. Other structural motifs include a transmembrane domain that anchors OPA1 in the mitochondrial inner membrane; the first coiled-coil domain, which is involved in protein-protein interactions; a GTPase domain crucial for protein activity; the middle domain, which participates in tetramerization and higher-order assembly of OPA1; and a second coiled-coil domain in the C-terminus (the GTPase effector domain, also called the assembly domain) that mediates the interaction between OPA1 and MFN1/2 [23] (Figure 1(b)).

Alternative splicing of exons 4, 4b, and 5 results in eight different isoforms of OPA1. Isoform 1 (exon 4) and isoform 7 (exons 4 and 5b) are the major expressed variants. To date, only two cleavage sites (located in exons 5 and 5b) have been detected in the primary *OPA1* sequence. Proteolysis at these sites leads to loss of the putative transmembrane domain and generation of the short soluble OPA1 isoform S-OPA1. 

Three types of mammalian proteases, presenilin-associated rhomboid-like protease (PARL), i-AAA metalloprotease (YmelL), and m-AAA metalloprotease (paraplegin), are the main regulators of OPA1 cleavage, although lack of these proteases does not seem to impair OPA1 processing [12, 55–57]. Although the contribution of PARL to OPA1 processing remains to be clarified, PARL seems to play an important role in generating S-OPA1, which controls the shape of mitochondrial cristae; cristae morphology is influenced by long OPA1 isoforms and by the amounts of S-OPA1 present [12, 13]. OPA1 downregulation causes cristae disorganization, whereas OPA1 overexpression narrows the mitochondrial cristae. In addition to its regulation by specific mitochondrial proteases, OPA1 can be processed under a loss of mitochondrial membrane potential. The mitochondrial inner-membrane metalloprotease OMA1 responds to low membrane potential and low levels of ATP through the proteolytic inactivation of OPA1. This mechanism prevents senescent or badly damaged mitochondria from undergoing inner-membrane fusion, isolating their matrix compartments and preventing contamination of the rest of the mitochondrial network. Other OPA1 post-translational modifications, ubiquitination and O-GlcNAcylation, have recently been documented in rat cells following exposure to prostaglandins or to a high-glucose environment [58, 59]. The impact of these modifications on OPA1 activity is largely unknown, and thus proteolytic processing remains the major pathway for achieving tight regulation of OPA1 functions.

The specific mechanism of OPA1-mediated mitochondrial fusion has not been fully elucidated, but as described in Song's report, both long and short OPA1 isoforms seem to be required for fusion to proceed efficiently, and balancing these isoforms requires the maintenance of inner-membrane potential [57, 60, 61]. Data reported by Ishihara et al. support a key role for L-OPA1 isoforms in fusion-stimulating activity; although Ishihara et al. demonstrated increased mitochondria fragmentation in S-isoform-expressing cells, further investigations should be carried out to gain better insight into the exact roles of the OPA1 isoforms [12].

The specific role of OPA3 in mitochondria dynamics needs to be clarified [62]. OPA3 maps to chromosome 19 and consists of three exons expressed in two transcripts, OPA3A and OPA3B. Both transcripts contain exon 1, which is spliced to exon 2 in OPA3A and to exon 3 in OPA3B. The encoded peptide is 179 amino acids long and displays a putative N-terminal mitochondrial signal sequence and a C-terminal coiled-coil domain. OPA3 is anchored to the mitochondrial outer membrane, with its N-terminus and C-terminus facing the intermembrane space and the cytoplasm, respectively. Ryu et al. observed that OPA3-overexpressing cells are more susceptible to apoptotic stimuli and that OPA3 triggers mitochondrial fragmentation, potentiating cell death through an FIS1/DRP1-independent pathway [62]. The 30 N-terminal residues of OPA3 are necessary for mitochondrial targeting, whereas the hydrophobic region is required for mitochondrial fragmentation [62]. Interaction between OPA3 and MFN1 has been suggested, although the underlying mechanism has not yet been identified. Mutations in the OPA3  hydrophobic region impair mitochondrial fragmentation and can induce an *MFN1* overexpression that leads to an imbalance between mitochondrial fusion and fission [62]. These observations could be the starting point for future studies of the physiopathological processes leading to *OPA3*-related disorders.

## 3. Human Disorders: *MFN2* Mutations Causing Charcot-Marie-Tooth (CMT) Disease Type 2A (CMT2A)

CMT disease is the most common inherited neuromuscular disorder, with a prevalence estimated at 1/2500, and it affects both motor and sensory neurons [63]. CMT is divided into two main categories according to electrophysiological criteria: demyelinating neuropathies with motor nerve conduction velocity <38 m/s (CMT1, 3, and 4) and axonal neuropathies with motor nerve conduction velocity >38 m/s [64]. Three main forms of axonal CMT diseases are classified according to genetic inheritance: dominant (CMT2), recessive (CMT4), and recessive X-linked (CMTX). CMT2A is the most prevalent type of axonal dominant form, occurring in up to 20% of all CMT2 patients, although recent findings of recessive or semidominant forms suggest a multiple inheritance pattern [65]. Mutations in *MFN2* are responsible for CMT2A and account for 20% of CMT, although one-quarter of these patients present with very mild CMT features [42, 66]. 

Typical clinical symptoms of CMT2A include progressive distal limb muscle weakness and atrophy, stepping gait with foot drop, and distal sensory loss. Mobility impairment can often be severe enough to cause wheelchair dependency. The sooner the symptoms appear, the more severe the disease course will be. Additional signs of CMT are the loss of deep tendon reflexes, usually associated with deformities such as a high arch or flexed toes, and (rarely) cranial nerve involvement, scoliosis, vocal cord paresis, or glaucoma. Central nervous system alterations and respiratory difficulties are very rare [67].

To date, more than 40 mutations affecting *MFN2* have been detected in patients with CMT2A (Mutation Database of Inherited Peripheral Neuropathies, http://www.molgen.ua.ac.be/cmtmutations/Home/Default.cfm/); most are missense mutations in the GTPase domain (>50%) or in the coiled-coil structure. The GTPase domain contains two hotspots in two highly conserved regions, one in the P-loop G1 subdomain (codon 94) and the second next to the G4 subdomain. Some MFN2 mutants show a gain of function and a tendency to aggregate in mitochondria, while others lose their function and thus impair mitochondrial fusion [68, 69].

It is remarkable that the same mutation can be associated with both early (before 10 years) and later onset of the disease, as well as with a range of CMT2A symptoms/signs that differ in severity among families in different countries [67]. Hence, a genotype/phenotype correlation has not yet been identified, suggesting that the environment or other modulatory genes are able to regulate the severity of the phenotype.

Interestingly, it was reported that defective mitochondrial fusion activity of *MFN2* mutants is rescued by overexpression of *MFN1*, which is consistent with the observation that MFN1 physically associates with MFN2 wild-type and CMT2A mutant forms to promote mitochondrial fusion [70, 71]. 

## 4. Human Disorders: *MFN2* Mutations Causing Other Hereditary Motor and Sensory Neuropathies (HMSNs)

A variety of additional symptoms are associated with several CMT forms, leading to the identification of new subtypes of HMSNs: type V, with spastic paraparesis, type VI, with optic atrophy, and type VII, with retinitis pigmentosa [72]. The typical clinical picture of CMT2A associated with pyramidal signs, which is classified as HMSN type V, was linked to mutations in *MFN2* inherited in an autosomal dominant pattern [43]. In affected patients, clinical onset usually occurs in the second decade of life or later, and disease progression is slow [72].

HMSN type VI exhibits both autosomal dominant and autosomal recessive traits, and its clinical features range from most severe to subclinical alterations, a range that is probably correlated to the age of onset [44, 73]. Typical clinical features consist of peripheral neuropathy and bilateral optic atrophy presenting with subacute visual loss, central scotomas, color-vision defects, and pale optic disks. Over the years, most patients experience various degrees of visual recovery similar to that observed in Lebers' hereditary optic neuropathy, although they can continue to show abnormal visual evoked potentials and bilateral optic disk pallor [44, 74]. Interestingly, no alterations were found in HMSN VI families screened for *OPA1*, the gene more strictly associated with optic atrophy [16, 17, 75], whereas defects and *de novo* mutations were identified in *MFN2* [44, 76].

## 5. Human Disorders: *OPA1* Mutations Causing ADOA

*OPA1* mutations lead to ADOA type 1, a neuroophthalmic disorder that typically starts during the first decade of life and is characterized by slowly progressive bilateral visual loss, dyschromatopsia, centrocecal scotomas, and temporal optic disk atrophy. The corresponding pathological hallmark is the selective degeneration of retinal ganglion cells. The incidence of ADOA ranges from 1/12000 to 1/50000. OPA1 mutations underlie approximately 60–70% of the cases of ADOA, also called Kjer's disease [77]. Most mutations in *OPA1* (approximately 50%) are predicted to lead to a truncated protein and to induce haploinsufficiency due to frameshift and nonsense mutations, stop codons, splicing errors, or deletions/insertions [78]. The remaining mutations are missense changes that cause heterozygous amino acid substitutions, approximately 30% of which preferentially occur within or close to the GTPase domain.

## 6. Human Disorders: *OPA3* Mutations Causing ADOA and Cataract (ADOAC) and Type III 3-Methylglutaconic Aciduria

ADOAC (OMIM 165300) was previously linked to mutations in *OPA3* [27]. In this instance, a premature blue cerulean cataract associated with optic neuropathy cosegregated in two French families as an autosomal dominant trait [27, 79]. Two dominant missense mutations were identified in *OPA3*: the c.277G>A transition in exon 2 and the c.313C>G transversion in exon 2 [27]. Fibroblasts from ADOAC patients exhibited no abnormalities in the respiratory chain, mitochondrial membrane potential, or morphology, although these cells were particularly susceptible to apoptosis [27].

Autosomal recessive *OPA3* mutations may also result in a progressive neurodegenerative disease featuring optic atrophy with 3-methylglutatonic aciduria [28]. Type III 3-methylglutaconic aciduria (OMIM 25801, also known as Costeff syndrome) is a rare neuroophthalmological syndrome characterized by increased urinary excretion of 3-methylglutaconic acid and 3-methylglutaric acid, early-onset bilateral optic atrophy, pallor of the optic disk, and reduced visual acuity, which are associated with spasticity, extrapyramidal signs, and cognitive deficits in later stages of the disease. The causative molecular defect of this condition was originally identified in a subset of Iraqi Jews [80]. Two homozygous mutations have been reported to date: the acceptor splice site variant c.143-1G>C and the in-frame deletion c.320_337del [28, 81]. The optic neuropathy present in carriers of *OPA3* mutations, which is typical of mitochondrial disorders, reflects OPA3's contribution to the control of mitochondrial homeostasis.

## 7. Human Disorders: *OPA1* and *MFN2* Mutations Causing mtDNA Deletions and Depletions

Over the years, several groups of nuclear genes have been shown to be involved in mtDNA maintenance. These genes encode essential parts of the mtDNA replicative/reparative machinery such as the mitochondrial DNA polymerase gamma, the hexameric helicase TWINKLE [82], and the exo/endonuclease flap-processing proteins MGME1 [83] and DNA2 [84]. Other gene products are involved in the mitochondrial nucleotide salvage pathway and maintenance of dNTP pools, including the ADP/ATP nucleotide translocator ANT1, deoxyguanosine kinase, and thymidine kinase. Deoxyguanosine kinase and thymidine kinase were previously associated with the severe infantile hepatocerebral and encephalomyopathic forms of mtDNA depletion, respectively, but recently these proteins have been reported to be responsible for milder clinical phenotypes in patients harboring multiple mtDNA deletions in skeletal muscle [85, 86]. Dominant-negative or gain-of-function mutations in RRMB2, which encodes the small subunit of the inducible ribonucleotide reductase p53R2, were previously reported in families affected with autosomal dominant PEO and accumulation of mtDNA deletions in postmitotic tissue [87].

The proteins involved in mitochondrial membrane fusion represent a further group of proteins whose alterations impair mtDNA homeostasis, since mutations in their coding genes lead to multisystemic pediatric- and adult-onset disorders, including PEO, with muscle accumulation of multiple mtDNA deletions or reduction of the amount of mtDNA [18, 46]. A subset of *OPA1* missense mutations have been associated with the “ADOA plus” syndrome and with accumulation of mtDNA deletions in stable tissues such as skeletal muscle. Incomplete penetrance (estimated to be as low as 43%), clinical variability, and age at onset ranging from birth to adulthood contribute to apparently sporadic ADOA cases [88]. 

It was previously reported that up to 20% of patients with *OPA1* mutations develop additional neuromuscular complications including deafness, ataxia, myopathy, peripheral neuropathy, and progressive external ophthalmoplegia [46]. A multiple sclerosis-like phenotype with white matter hyperintense signals in fluid attenuated inversion recovery studies was also reported [45, 88]. In this case, the clinical picture was characterized by optic nerve involvement and gait alterations such as ataxia, which were associated with typical findings in radiological and laboratory investigations. Patients  harboring *OPA1* mutations can also present progressive spastic paraparesis in lower limbs followed by optic neuropathy and sensorimotor neuropathy [46].

In the ADOA and ADOA plus phenotypes, muscle biopsies often do not reveal hallmarks of mitochondrial myopathy, whereas investigation by phosphorus-31 magnetic resonance spectroscopy demonstrated defective oxidative metabolism and a reduced rate of ATP synthesis in skeletal muscle [16, 89, 90]. Hence, additional unknown factors may contribute to the clinical manifestations of *OPA1* mutations [90].

Similar complex phenotypes have also been associated with *MFN2* mutations. Optic atrophy beginning in early childhood followed by adult-onset axonal neuropathy and mitochondrial myopathy without external ophthalmoplegia was the main clinical features described in a Tunisian family carrying the novel *MFN2* missense mutation c.629A>T (p.D210V). Muscle analysis revealed typical aspects of mitochondrial stress, such as ragged red and COX-negative fibers and an accumulation of mtDNA deletions [18] (Table 2). The implication of *MFN2* in mtDNA maintenance, replication, and repair is indirectly supported by the qualitative (multiple deletions) and quantitative (depletion) abnormalities of mtDNA molecules in the skeletal muscle of mitofusin-deficient mice [19]. Studies performed in *MFN2* knockout mouse muscles suggested a correlation between the impairment of mitochondrial fusion and compensatory mitochondrial proliferation in both interfibrillar and subsarcolemmal spaces, muscle atrophy, and defective mtDNA content and integrity [19, 91].

Alteration of the mitochondrial fusion pathway is also associated with mtDNA depletion syndromes [47], which are usually devastating diseases of infancy or early childhood with autosomal recessive inheritance; these syndromes are characterized by decreased mtDNA content in postmitotic tissues and result in severe fatal myopathic, encephalomyopathic, or hepatocerebral syndromes [92]. A reduction in mtDNA copy number has been reported in the muscles of patients carrying *MFN2* mutations, who manifested milder phenotypes with onset of clinical symptoms occurring from childhood through adulthood. Various degrees of gait impairment, decline in visual acuity, and central nervous system involvement have been reported. Histological analysis and electron microscopy of muscle biopsies revealed some COX-deficient fibers and a paucity of intermyofibrillar mitochondria, which appeared swollen and smaller in size [47].

## 8. Mitochondrial Fusion/Fission and Other Neurodegenerative Disorders

Recent evidence supports the growing importance of mitochondrial fission and fusion proteins in cell survival and in antiapoptotic pathways, which contribute, directly or indirectly, to the etiopathogenesis of several human disorders. Mitochondrial dysfunction was previously associated with rare neurodegenerative disorders such as CMT2A and ADOA as well as more common disorders, including Alzheimer's, Huntington's, and Parkinson's diseases [48, 93–96]. Physiologically, mitochondrial functions deteriorate over the years due to the accumulation of pathogenic alterations in mtDNA, the slow decline of respiratory chain activity, and an imbalance between the production of reactive oxygen species and antioxidant mechanisms [97, 98]. Senescent mitochondria exhibit morphological and functional alterations similar to those observed in neurodegenerative disease [48, 93, 99–101].

Studies performed on tissues and cells collected from Alzheimer's disease patients revealed an imbalance in fusion and fission that resulted in increased mitochondrial fragmentation. In particular, reduced levels of MFN1, MFN2, and OPA1 and high levels of FIS1 and DLP1/DRP1 were observed. Notably, FIS1 and DLP1 displayed aberrant interactions with the Alzheimer's disease-related proteins *β*-amyloid and phosphorylated tau [99–101].

The causative role of altered mitochondrial dynamics in Parkinson's disease seems to be a mature and established hypothesis. In familial juvenile forms of Parkinson's disease due to recessive mutations in mitophagy-related genes such as Parkin and PINK1, several lines of evidence point toward direct impairment of mitochondrial fission/fusion pathways [102, 103]. The Parkin-PINK 1 axis plays a regulating role in mitochondrial quality control, participating in MFN2 ubiquitination and degradation [104]. Further, selective *MFN2* ablation in a conditional mouse model caused the degeneration of axonal projections of dopaminergic neurons to the striatum prior to the death of soma, suggesting a “dying-back” type of neurodegeneration [48, 105]. Conversely, *MFN1*-null mice developed neither locomotive defects nor impairment in the nigrostriatal circuit [48, 106]. Further studies are required to address the complex molecular mechanisms underlying these neurodegenerative diseases and to modify the courses of these diseases via new therapeutic targets.

Glutamate-mediated excitotoxic damage is also known to participate in neurodegeneration.* OPA1* was previously shown to play a central role in the response to excitotoxic cellular insult induced by overactivation of *N*-methyl-D-aspartate (NMDA) receptors via altered mitochondrial calcium homeostasis [107–110]. Overactivation of NMDA receptors increases mitochondrial fragmentation, arrests mitochondrial fusion, and causes several ultrastructural defects in the inner mitochondrial membrane, such as a widening of intercristal distances [111]. Since OPA1 is the major regulator of cristae morphology, Jahani-Asl and colleagues explored its role in glutamate-mediated cellular damage. They demonstrated that *OPA1* overexpression played a protective role against NMDA-mediated excitotoxic neuronal damage, resulting in increased survival of neuronal cells and in the rescue of mitochondrial morphology [111]. OPA1 seemed to share a functional pathway with calpastatin, an inhibitor of the calcium-dependent protease calpain, which is involved in apoptosis [111]. Further experiments confirmed an increased sensibility to glutamate-mediated damage of retinal cells in both mouse and cellular models in which *OPA1* expression was knocked down [110, 112]. Therefore, this mechanism may promote the degeneration of retinal cells in ADOA. In addition, since OPA1 expression is reduced in other neurodegenerative disorders, a similar etiopathogenetic pathway can be hypothesized.

## Figures and Tables

**Table 1:** Clinical features and their prevalence according to mutation.

Clinical features	MFN2	OPA1	OPA3	PINK1
Axonal neuropathy	+++	+		
Optic atrophy	+	+++	+++	
Deafness	+	++		
Limb girdle weakness	+	+		
Progressive external ophthalmoplegia	++	++		
Cataract			+++	
Resting tremor, rigidity, and bradykinesia				+++
White matter periventricular involvement	+	+		
3-Methylglutaconic aciduria			+++	
Cognitive decline	+	+	+	+
Spasticity	++		++	
Ataxia	+	+		

+++: typical; ++: common; +: infrequent.

**Table 2:** Clinical phenotype variability associated with mutations in mitofusin 2.

Phenotype	Reference
Charcot-Marie-Tooth type 2A	[42]
Hereditary motor and sensory neuropathy, type V	[43]
Hereditary motor and sensory neuropathy, type VI	[44]
Multiple sclerosis-like disorder	[45]
Autosomal dominant optic atrophy plus and mitochondrial DNA multiple deletions syndrome	[18, 46]
Mitochondrial DNA depletion syndrome	[47]
Parkinson's disease	[48]

## References

[1] Youle RJ, van der Bliek AM (2012). Mitochondrial fission, fusion, and stress. *Science*.

[2] Yoneda M, Miyatake T, Attardi G (1994). Complementation of mutant and wild-type human mitochondrial DNAs coexisting since the mutation event and lack of complementation of DNAs introduced separately into a cell within distinct organelles. *Molecular and Cellular Biology*.

[3] Nakada K, Inoue K, Ono T (2001). Inter-mitochondrial complementation: mitochondria-specific system preventing mice from expression of disease phenotypes by mutant mtDNA. *Nature Medicine*.

[4] Milone M, Benarroch EE (2012). Mitochondrial dynamics: general concepts and clinical implications. *Neurology*.

[5] Smirnova E, Griparic L, Shurland DL, Van der Bliek AM (2001). Dynamin-related protein Drp1 is required for mitochondrial division in mammalian cells. *Molecular Biology of the Cell*.

[6] Otera H, Wang C, Cleland MM (2010). Mff is an essential factor for mitochondrial recruitment of Drp1 during mitochondrial fission in mammalian cells. *Journal of Cell Biology*.

[7] Palmer CS, Osellame LD, Laine D, Koutsopoulos OS, Frazier AE, Ryan MT (2011). MiD49 and MiD51, new components of the mitochondrial fission machinery. *EMBO Reports*.

[8] De Brito OM, Scorrano L (2008). Mitofusin 2 tethers endoplasmic reticulum to mitochondria. *Nature*.

[9] Darshi M, Mendiola VL, Mackey MR (2011). ChChd3, an inner mitochondrial membrane protein, is essential for maintaining Crista integrity and mitochondrial function. *Journal of Biological Chemistry*.

[10] Cipolat S, Martins DB, Dal Zilio B, Scorrano L (2004). OPA1 requires mitofusin 1 to promote mitochondrial fusion. *,Proceedings of the National Academy of Sciences of the United States of America*.

[11] Griparic L, Van Der Wel NN, Orozco IJ, Peters PJ, Van Der Bliek AM (2004). Loss of the intermembrane space protein Mgm1/OPA1 induces swelling and localized constrictions along the lengths of mitochondria. *Journal of Biological Chemistry*.

[12] Ishihara N, Fujita Y, Oka T, Mihara K (2006). Regulation of mitochondrial morphology through proteolytic cleavage of OPA1. *EMBO Journal*.

[13] Olichon A, Baricault L, Gas N (2003). Loss of OPA1 perturbates the mitochondrial inner membrane structure and integrity, leading to cytochrome c release and apoptosis. *Journal of Biological Chemistry*.

[14] Song Z, Ghochani M, McCaffery JM, Frey TG, Chan DC (2009). Mitofusins and OPA1 mediate sequential steps in mitochondrial membrane fusion. *Molecular Biology of the Cell*.

[15] Palmer CS, Osellame LD, Stojanovski D, Ryan MT (2011). The regulation of mitochondrial morphology: intricate mechanisms and dynamic machinery. *Cellular Signalling*.

[16] Amati-Bonneau P, Valentino ML, Reynier P (2008). OPA1 mutations induce mitochondrial DNA instability and optic atrophy “plus” phenotypes. *Brain*.

[17] Hudson G, Amati-Bonneau P, Blakely EL (2008). Mutation of OPA1 causes dominant optic atrophy with external ophthalmoplegia, ataxia, deafness and multiple mitochondrial DNA deletions: a novel disorder of mtDNA maintenance. *Brain*.

[18] Rouzier C, Bannwarth S, Chaussenot A (2012). The MFN2 gene is responsible for mitochondrial DNA instability and optic atrophy “plus” phenotype. *Brain*.

[19] Chen H, Vermulst M, Wang YE (2010). Mitochondrial fusion is required for mtdna stability in skeletal muscle and tolerance of mtDNA mutations. *Cell*.

[20] Hori A, Yoshida M, Ling F (2011). Mitochondrial fusion increases the mitochondrial DNA copy number in budding yeast. *Genes to Cells*.

[21] Bach D, Pich S, Soriano FX (2003). Mitofusin-2 determines mitochondrial network architecture and mitochondrial metabolism. A novel regulatory mechanism altered in obesity. *Journal of Biological Chemistry*.

[22] Chen KH, Guo X, Ma D (2006). Dysregulation of HSG triggers vascular proliferative disorders. *Nature Cell Biology*.

[23] Zorzano A, Liesa M, Sebastián D (2010). Mitochondrial fusion proteins: dual regulators of morphology and metabolism. *Seminars in Cell & Developmental Biology*.

[24] Papanicolaou KN, Khairallah RJ, Ngoh GA (2011). Mitofusin-2 maintains mitochondrial structure and contributes to stress-induced permeability transition in cardiac myocytes. *Molecular and Cellular Biology*.

[25] Chen Y, Liu Y, Dorn II GW (2011). Mitochondrial fusion is essential for organelle function and cardiac homeostasis. *Circulation Research*.

[26] Chen L, Liu T, Tran A (2012). OPA1 mutation and late-onset cardiomyopathy: mitochondrial dysfunction and mtDNA instability. *Journal of the American Heart Association*.

[27] Reynier P, Amati-Bonneau P, Verny C (2004). OPA3 gene mutations responsible for autosomal dominant optic atrophy and cataract. *Journal of Medical Genetics*.

[28] Anikster Y, Kleta R, Shaag A, Gahl WA, Elpeleg O (2001). Type III 3-methylglutaconic aciduria (optic atrophy plus syndrome, or costeff optic atrophy syndrome): identification of the OPA3 gene and its founder mutation in Iraqi Jews. *American Journal of Human Genetics*.

[29] Huizing M, Dorward H, Ly L (2010). OPA3, mutated in 3-methylglutaconic aciduria type III, encodes two transcripts targeted primarily to mitochondria. *Molecular Genetics and Metabolism*.

[30] Thomas DY, Wilkie D (1968). Recombination of mitochondrial drug-resistance factors in Saccharomyces, cerevisiae. *Biochemical and Biophysical Research Communications*.

[31] Nunnari J, Marshall WF, Straight A, Murray A, Sedat JW, Walter P (1997). Mitochondrial transmission during mating in Saccharomyces cerevisiae is determined by mitochondrial fusion and fission and the intramitochondrial segregation of mitochondrial DNA. *Molecular Biology of the Cell*.

[32] Bereiter-Hahn J, Voth M (1994). Dynamics of mitochondria in living cells: shape changes, dislocations, fusion, and fission of mitochondria. *Microscopy Research and Technique*.

[33] Cortese JD, Voglino LA, Hackenbrock CR (1998). Novel fluorescence membrane fusion assays reveal GTP-dependent fusogenic properties of outer mitochondrial membrane-derived proteins. *Biochimica et Biophysica Acta*.

[34] Hales KG, Fuller MT (1997). Developmentally regulated mitochondrial fusion mediated by a conserved, novel, predicted GTPase. *Cell*.

[35] Yaffe MP (1999). The machinery of mitochondrial inheritance and behavior. *Science*.

[36] Bleazard W, McCaffery JM, King EJ (1999). The dynamin-related GTPase Dnm1 regulates mitochondrial fission in yeast. *Nature Cell Biology*.

[37] Labrousse AM, Zappaterra MD, Rube DA, Van der Bliek AM (1999). C. elegans dynamin-related protein DRP-1 controls severing of the mitochondrial outer membrane. *Molecular Cell*.

[38] Shin HW, Shinotsuka C, Torii S, Murakami K, Nakayama K (1997). Identification and subcellular localization of a novel mammalian dynamin-related protein homologous to yeast Vps1p and Dnm1p. *Journal of Biochemistry*.

[39] Imoto M, Tachibana I, Urrutia R (1998). Identification and functional characterization of a novel human protein highly related to the yeast dynamin-like GTPase Vps1p. *Journal of Cell Science*.

[40] Meeusen S, McCaffery JM, Nunnari J (2004). Mitochondrial fusion intermediates revealed in vitro. *Science*.

[41] Meeusen SL, Nunnari J (2007). Mitochondrial fusion in vitro. *Methods in Molecular Biology*.

[42] Züchner S, Mersiyanova IV, Muglia M (2004). Mutations in the mitochondrial GTPase mitofusin 2 cause Charcot-Marie-Tooth neuropathy type 2A. *Nature Genetics*.

[43] Zhu D, Kennerson ML, Walizada G, Züchner S, Vance JM, Nicholson GA (2005). Charcot-Marie-Tooth with pyramidal signs is genetically heterogeneous: families with and without MFN2 mutations. *Neurology*.

[44] Züchner S, De Jonghe P, Jordanova A (2006). Axonal neuropathy with optic atrophy is caused by mutations in mitofusin 2. *Annals of Neurology*.

[45] Verny C, Loiseau D, Scherer C (2008). Multiple sclerosis-like disorder in OPA1-related autosomal dominant optic atrophy. *Neurology*.

[46] Yu-Wai-Man P, Griffiths PG, Gorman GS (2010). Multi-system neurological disease is common in patients with OPA1 mutations. *Brain*.

[47] Vielhaber S, Debska-Vielhaber G, Peeva V (2013). Mitofusin 2 mutations affect mitochondrial function by mitochondrial DNA depletion. *Acta Neuropathologica*.

[48] Lee S, Sterky FH, Mourier A (2012). Mitofusin 2 is necessary for striatal axonal projections of midbrain dopamine neurons. *Human Molecular Genetics*.

[49] Ishihara N, Otera H, Oka T, Mihara K Regulation and physiologic functions of GTPases in mitochondrial fusion and fission in mammals.

[50] Anton F, Fres JM, Schauss A (2011). Ugo1 and Mdm30 act sequentially during Fzo1-mediated mitochondrial outer membrane fusion. *Journal of Cell Science*.

[51] Cohen MM, Amiott EA, Day AR (2011). Sequential requirements for the GTPase domain of the mitofusin Fzo1 and the ubiquitin ligase SCFMdm30 in mitochondrial outer membrane fusion. *Journal of Cell Science*.

[52] Tanaka A, Cleland MM, Xu S (2010). Proteasome and p97 mediate mitophagy and degradation of mitofusins induced by Parkin. *Journal of Cell Biology*.

[53] Koshiba T, Detmer SA, Kaiser JT, Chen H, McCaffery JM, Chan DC (2004). Structural basis of mitochondrial tethering by mitofusin complexes. *Science*.

[54] Huang P, Galloway CA, Yoon Y (2011). Control of mitochondrial morphology through differential interactions of mitochondrial fusion and fission proteins. *PLoS ONE*.

[55] Duvezin-Caubet S, Koppen M, Wagener J (2007). OPA1 processing reconstituted in yeast depends on the subunit composition of the m-AAA protease in mitochondria. *Molecular Biology of the Cell*.

[56] Pellegrini L, Passer BJ, Canelles M (2001). PAMP and PARL, two novel putative metalloproteases interacting with the COOH-terminus of Presenilin-1 and -2. *Journal of Alzheimer’s Disease*.

[57] Song Z, Chen H, Fiket M, Alexander C, Chan DC (2007). OPA1 processing controls mitochondrial fusion and is regulated by mRNA splicing, membrane potential, and Yme1L. *Journal of Cell Biology*.

[58] Kar R, Mishra N, Singha PK, Venkatachalam MA, Saikumar P (2010). Mitochondrial remodeling following fission inhibition by 15d-PGJ2 involves molecular changes in mitochondrial fusion protein OPA1. *Biochemical and Biophysical Research Communications*.

[59] Makino A, Suarez J, Gawlowski T (2011). Regulation of mitochondrial morphology and function by O-GlcNAcylation in neonatal cardiac myocytes. *American Journal of Physiology*.

[60] Ishihara N, Jofuku A, Eura Y, Mihara K (2003). Regulation of mitochondrial morphology by membrane potential, and DRP1-dependent division and FZO1-dependent fusion reaction in mammalian cells. *Biochemical and Biophysical Research Communications*.

[61] Legros F, Lombès A, Frachon P, Rojo M (2002). Mitochondrial fusion in human cells is efficient, requires the inner membrane potential, and is mediated by mitofusins. *Molecular Biology of the Cell*.

[62] Ryu SW, Jeong HJ, Choi M, Karbowski M, Choi C (2010). Optic atrophy 3 as a protein of the mitochondrial outer membrane induces mitochondrial fragmentation. *Cellular and Molecular Life Sciences*.

[63] Vance JM (2000). The many faces of Charcot-Marie-Tooth disease. *Archives of Neurology*.

[64] Harding AE, Thomas PK (1980). The clinical features of hereditary motor and sensory neuropathy types I and II. *Brain*.

[65] Polke JM, Laurá M, Pareyson D (2011). Recessive axonal Charcot-Marie-Tooth disease due to compound heterozygous mitofusin 2 mutations. *Neurology*.

[66] Kijima K, Numakura C, Izumino H (2005). Mitochondrial GTPase mitofusin 2 mutation in Charcot-Marie-Tooth neuropathy type 2A. *Human Genetics*.

[67] Chung KW, Kim SB, Park KD (2006). Early onset severe and late-onset mild Charcot-Marie-Tooth disease with mitofusin 2 (MFN2) mutations. *Brain*.

[68] Verhoeven K, Claeys KG, Züchner S (2006). MFN2 mutation distribution and genotype/phenotype correlation in Charcot-Marie-Tooth type 2. *Brain*.

[69] Calvo J, Funalot B, Ouvrier RA (2009). Genotype-phenotype correlations in Charcot-Marie-Tooth disease type 2 caused by mitofusin 2 mutations. *Archives of Neurology*.

[70] Amiott EA, Lott P, Soto J (2008). Mitochondrial fusion and function in Charcot-Marie-Tooth type 2A patient fibroblasts with mitofusin 2 mutations. *Experimental Neurology*.

[71] Detmer SA, Chan DC (2007). Complementation between mouse Mfn1 and Mfn2 protects mitochondrial fusion defects caused by CMT2A disease mutations. *Journal of Cell Biology*.

[72] Dyck PJ, Chance P, Lebo R, Carney JA, Dyck PJ, Thomas PK (1993). Hereditary motor and sensory neuropathy. *Peripheral Neuropathy*.

[73] Chalmers RM, Bird AC, Harding AE (1996). Autosomal dominant optic atrophy with asymptomatic peripheral neuropathy. *Journal of Neurology Neurosurgery and Psychiatry*.

[74] Huoponen K (2001). Leber hereditary optic neuropathy: clinical and molecular genetic findings. *Neurogenetics*.

[75] Alexander C, Votruba M, Pesch UEA (2000). OPA1, encoding a dynamin-related GTPase, is mutated in autosomal dominant optic atrophy linked to chromosome 3q28. *Nature Genetics*.

[76] Voo I, Allf BE, Udar N, Silva-Garcia R, Vance J, Small KW (2003). Hereditary motor and sensory neuropathy type VI with optic atrophy. *American Journal of Ophthalmology*.

[77] Kjer P (1959). Infantile optic atrophy with dominant mode of inheritance: a clinical and genetic study of 19 Danish families. *Acta Ophthalmologica*.

[78] Ferré M, Amati-Bonneau P, Tourmen Y, Malthièry Y, Reynier P (2005). eOPA1: an online database for OPA1 mutations. *Human Mutation*.

[79] Garcin R, Raverdy P, Delthil S (1961). On a heredo-familial disease combining cataract, optic atrophy, extrapyramidal symptoms and certain defects of Friedreich's disease. (Its nosological position in relation to the Behr's syndrome, the Marinesco-Sjogren syndrome and Friedreich's disease with ocular symptoms. *Revue Neurologique*.

[80] Nystuen A, Costeff H, Elpeleg ON (1997). Iraqi-Jewish kindreds with optic atrophy plus (3-methylglutaconic aciduria type 3) demonstrate linkage disequilibrium with the CTG repeat in the 3’ untranslated region of the myotonic dystrophy protein kinase gene. *Human Molecular Genetics*.

[81] Kleta R, Skovby F, Christensen E, Rosenberg T, Gahl WA, Anikster Y (2002). 3-Methylglutaconic aciduria type III in a non-Iraqi-Jewish kindred: clinical and molecular findings. *Molecular Genetics and Metabolism*.

[82] Hudson G, Chinnery PF (2006). Mitochondrial DNA polymerase-*γ* and human disease. *Human Molecular Genetics*.

[83] Kornblum C, Nicholls TJ, Haack TB (2013). Loss-of-function mutations in MGME1 impair mtDNA replication and cause multisystemic mitochondrial disease. *Nature Genetics*.

[84] Ronchi D, Di Fonzo A, Lin W (2013). Mutations in DNA2 link progressive myopathy to mitochondrial DNA instability. *American Journal of Human Genetics*.

[85] Ronchi D, Garone C, Bordoni A (2012). Next-generation sequencing reveals DGUOK mutations in adult patients with mitochondrial DNA multiple deletions. *Brain*.

[86] Tyynismaa H, Sun R, Ahola-Erkkilä S (2012). Thymidine kinase 2 mutations in autosomal recessive progressive external ophthalmoplegia with multiple mitochondrial DNA deletions. *Human Molecular Genetics*.

[87] Tyynismaa H, Ylikallio E, Patel M, Molnar MJ, Haller RG, Suomalainen A (2009). A heterozygous truncating mutation in RRM2B causes autosomal-dominant progressive external ophthalmoplegia with multiple mtDNA deletions. *American Journal of Human Genetics*.

[88] Amati-Bonneau P, Milea D, Bonneau D (2009). OPA1-associated disorders: phenotypes and pathophysiology. *International Journal of Biochemistry & Cell Biology*.

[89] Lodi R, Tonon C, Valentino ML (2011). Defective mitochondrial adenosine triphosphate production in skeletal muscle from patients with dominant optic atrophy due to OPA1 mutations. *Archives of Neurology*.

[90] Yu-Wai-Man P, Trenell MI, Hollingsworth KG, Griffiths PG, Chinnery PF (2011). OPA1 mutations impair mitochondrial function in both pure and complicated dominant optic atrophy. *Brain*.

[91] Sitarz KS, Yu-Wai-Man P, Pyle A (2012). MFN2 mutations cause compensatory mitochondrial DNA proliferation. *Brain*.

[92] Ricci E, Moraes CT, Servidei S, Tonali P, Bonilla E, DiMauro S (1992). Disorders associated with depletion of mitochondrial DNA. *Brain Pathology*.

[93] Santos D, Cardoso SM (2012). Mitochondrial dynamics and neuronal fate in Parkinson's disease. *Mitochondrion*.

[94] Cho DH, Nakamura T, Lipton SA (2010). Mitochondrial dynamics in cell death and neurodegeneration. *Cellular and Molecular Life Sciences*.

[95] Kim J, Moody JP, Edgerly CK (2010). Mitochondrial loss, dysfunction and altered dynamics in Huntington’s disease. *Human Molecular Genetics*.

[96] Reddy PH, Shirendeb UP (2012). Mutant huntingtin, abnormal mitochondrial dynamics, defective axonal transport of mitochondria, and selective synaptic degeneration in Huntington’s disease. *Biochimica et Biophysica Acta*.

[97] Larsson NG (2010). Somatic mitochondrial DNA mutations in mammalian aging. *Annual Review of Biochemistry*.

[98] Wallace DC (2010). Mitochondrial DNA mutations in disease and aging. *Environmental and Molecular Mutagenesis*.

[99] Wang X, Su B, Lee HG (2009). Impaired balance of mitochondrial fission and fusion in Alzheimer’s disease. *Journal of Neuroscience*.

[100] Manczak M, Reddy PH (2012). Abnormal interaction between the mitochondrial fission protein Drp1 and hyperphosphorylated tau in Alzheimer’s disease neurons: implications for mitochondrial dysfunction and neuronal damage. *Human Molecular Genetics*.

[101] Manczak M, Calkins MJ, Reddy PH (2011). Impaired mitochondrial dynamics and abnormal interaction of amyloid beta with mitochondrial protein Drp1 in neurons from patients with Alzheimer’s disease: implications for neuronal damage. *Human Molecular Genetics*.

[102] Youle RJ, Narendra DP (2011). Mechanisms of mitophagy. *Nature Reviews Molecular Cell Biology*.

[103] Kitada T, Asakawa S, Hattori N (1998). Mutations in the parkin gene cause autosomal recessive juvenile parkinsonism. *Nature*.

[104] Gegg ME, Cooper JM, Chau KY, Rojo M, Schapira AHV, Taanman JW (2010). Mitofusin 1 and mitofusin 2 are ubiquitinated in a PINK1/parkin-dependent manner upon induction of mitophagy. *Human Molecular Genetics*.

[105] Misko AL, Sasaki Y, Tuck E (2012). Mitofusin2 mutations disrupt axonal mitochondrial positioning and promote axon degeneration. *Journal of Neuroscience*.

[106] Pham AH, Meng S, Chu QN, Chan DC (2012). Loss of Mfn2 results in progressive, retrograde degeneration of dopaminergic neurons in the nigrostriatal circuit. *Human Molecular Genetics*.

[107] Manev H, Favaron M, Guidotti A, Costa E (1989). Delayed increase of Ca2+ influx elicited by glutamate: role in neuronal death. *Molecular Pharmacology*.

[108] Randall RD, Thayer SA (1992). Glutamate-induced calcium transient triggers delayed calcium overload and neurotoxicity in rat hippocampal neurons. *Journal of Neuroscience*.

[109] Budd SL, Nicholls DG (1996). Mitochondria, calcium regulation, and acute glutamate excitotoxicity in cultured cerebellar granule cells. *Journal of Neurochemistry*.

[110] Kushnareva YE, Gerencser AA, Bossy B (2013). Loss of OPA1 disturbs cellular calcium homeostasis and sensitizes for excitotoxicity. *Cell Death & Differentiation*.

[111] Jahani-Asl A, Pilon-Larose K, Xu W (2011). The mitochondrial inner membrane GTPase, optic atrophy 1 (Opa1), restores mitochondrial morphology and promotes neuronal survival following excitotoxicity. *Journal of Biological Chemistry*.

[112] Nguyen D, Alavi MV, Kim T KY (2011). A new vicious cycle involving glutamate excitotoxicity, oxidative stress and mitochondrial dynamics. *Cell Death and Disease*.

